# Supplementary material for: Antibody Mediated Intercommunication of Germinal Centers
Source: Cells. 2022 Nov 19;11(22):3680. doi: 10.3390/cells11223680 (PMC9688628; doi:10.3390/cells11223680)
Supplement: Supplementary file 1 [file cells-11-03680-s001.zip › cells-1856857-supplementary.pdf]

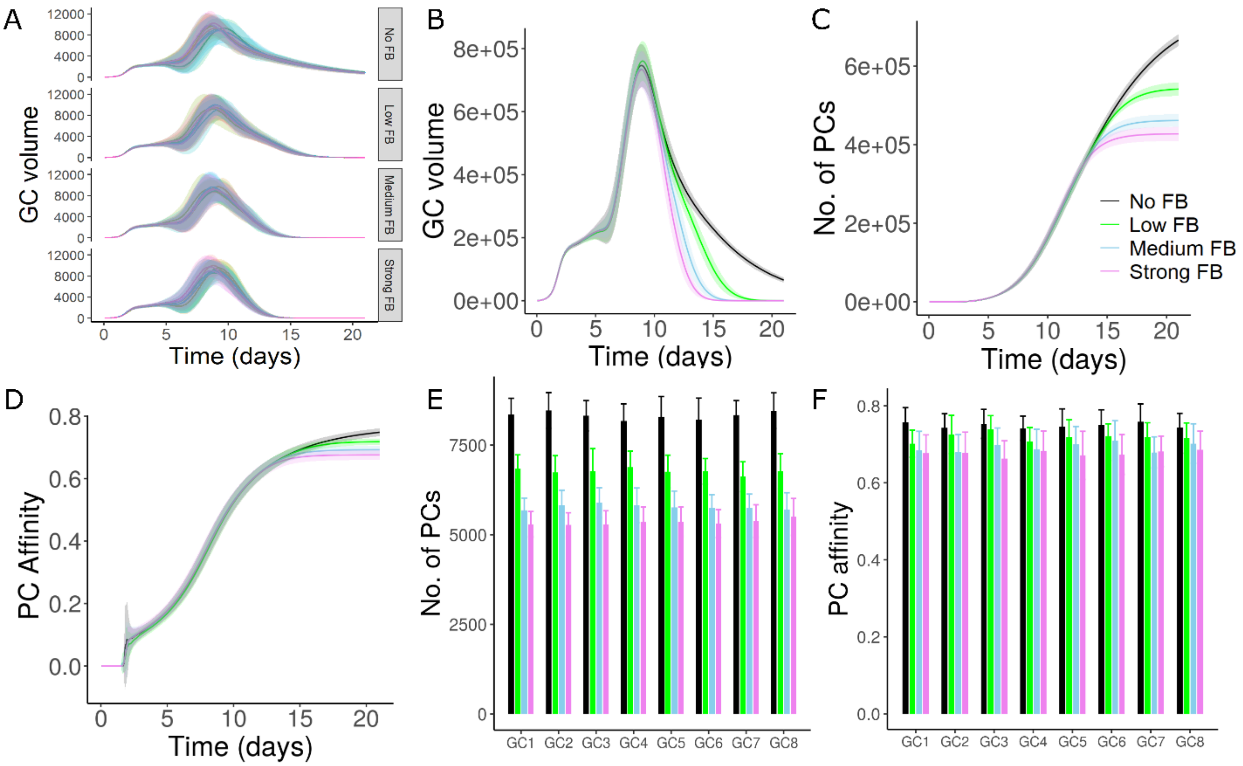

Figure S1: Impact of GC-GC interactions as in Figure 1 but with synchronous GC onset. A) Volume kinetics of each simulated GC. Color code shows individual GCs. B) Total volume of all GCs. C) Total plasma cell production from all GCs. D) Affinity of plasma cells from all GCs. Readouts in B-D represent the sum of corresponding readouts of all simulated GCs weighted by the number of non-explicitly simulated GCs in each case. E) Number of plasma cells produced from each simulated GC. F) Affinity of plasma cells from each simulated GC. Inset in panel C shows the GC-GC interaction strength and applies for panels B-F. A single epitope (shape space position 3333) was considered and founder cell positions were chosen randomly anywhere in the shape space.

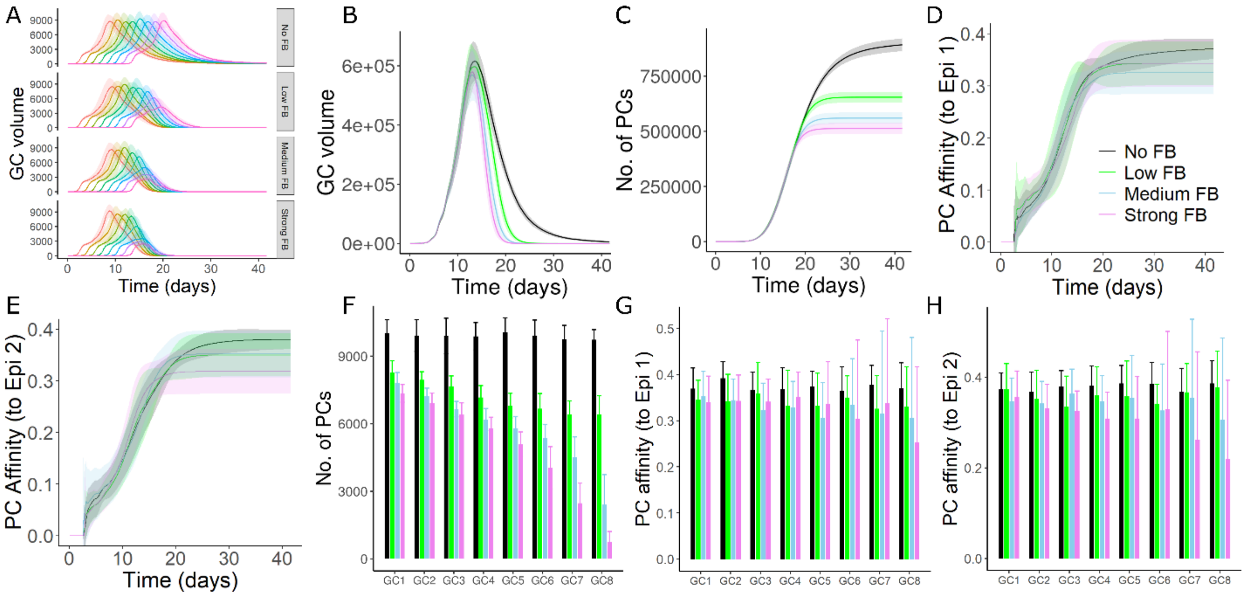

Figure S2: Impact of GC-GC interactions as in Figure 1 but with two epitopes in equal proportions. A) Volume kinetics of simulated GCs. Color code represents the timing of GC initiation. B) Total volume of all GCs. C) Total plasma cell production of all GCs. D) Affinity of plasma cells from all GCs to Epi 1. E) Affinity of plasma cells from all GCs to Epi 2. Readouts in B-E represent the sum of corresponding readouts of all simulated GCs weighted by the number of non-explicitly simulated GCs in each case. F-H) Number of plasma cells, affinity of plasma cells to Epi 1 and 2 from each simulated GC. GCs were initialized asynchronously with two epitopes in equal proportion (shape space positions 3333 and 5555) and founder cell positions chosen randomly anywhere in the shape space. GCs 1-8 were sorted in the sequence of initiation. Inset in panel D shows the GC-GC interaction strength and applies for panels B-H.

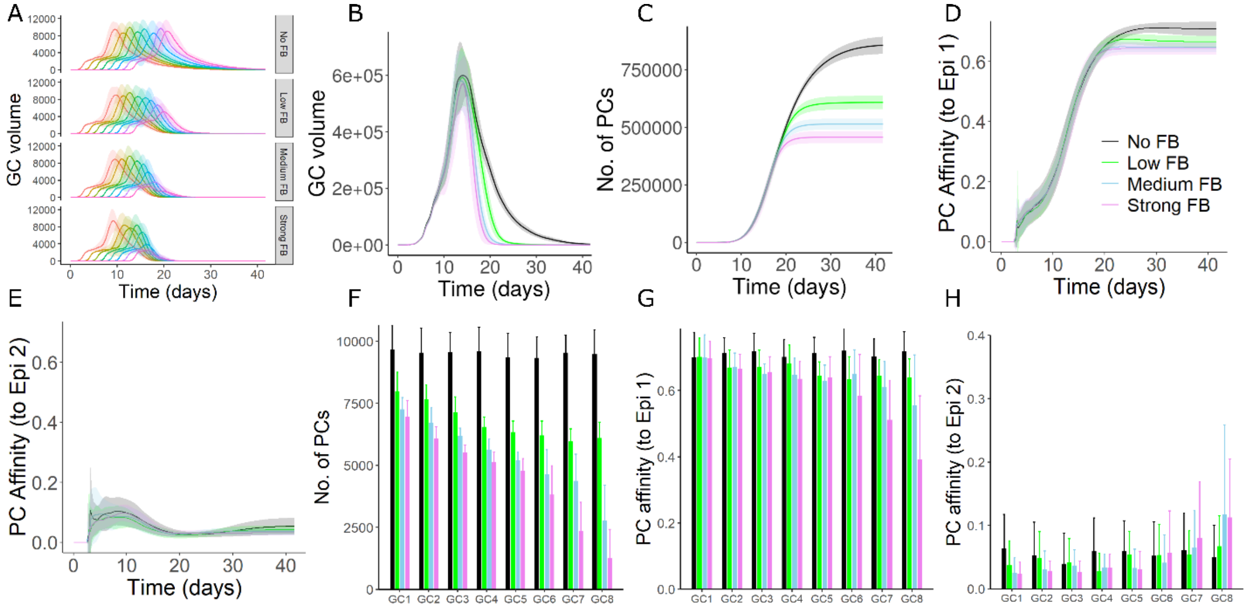

Figure S3: Impact of GC-GC interactions as in Figure 1 but with an immunodominant (Epi 1) and rare epitope (Epi 2). A) Volume kinetics of simulated GCs. GCs were initialized asynchronously and color code represents the timing of GC initiation. B) Total volume of all GCs. C) Total plasma cell production of all GCs. D) Affinity of plasma cells from all GCs to Epi 1.E) Affinity of plasma cells from all GCs to Epi 2. Readouts in B-E represent the sum of corresponding readouts of all simulated GCs weighted by the number of non-explicitly simulated GCs in each case. F-H) Number of plasma cells, affinity of plasma cells to Epi 1 and 2 from each simulated GC. Two epitopes were considered in unequal proportion 90% (Epi 1; shape space position 3333) and 10 % (Epi 2; shape space position 5555). Founder cells were chosen randomly anywhere in the shape space. GCs 1-8 were sorted in the sequence of initiation. Inset in panel D shows the GC-GC interaction strength and applies for panels B-H.

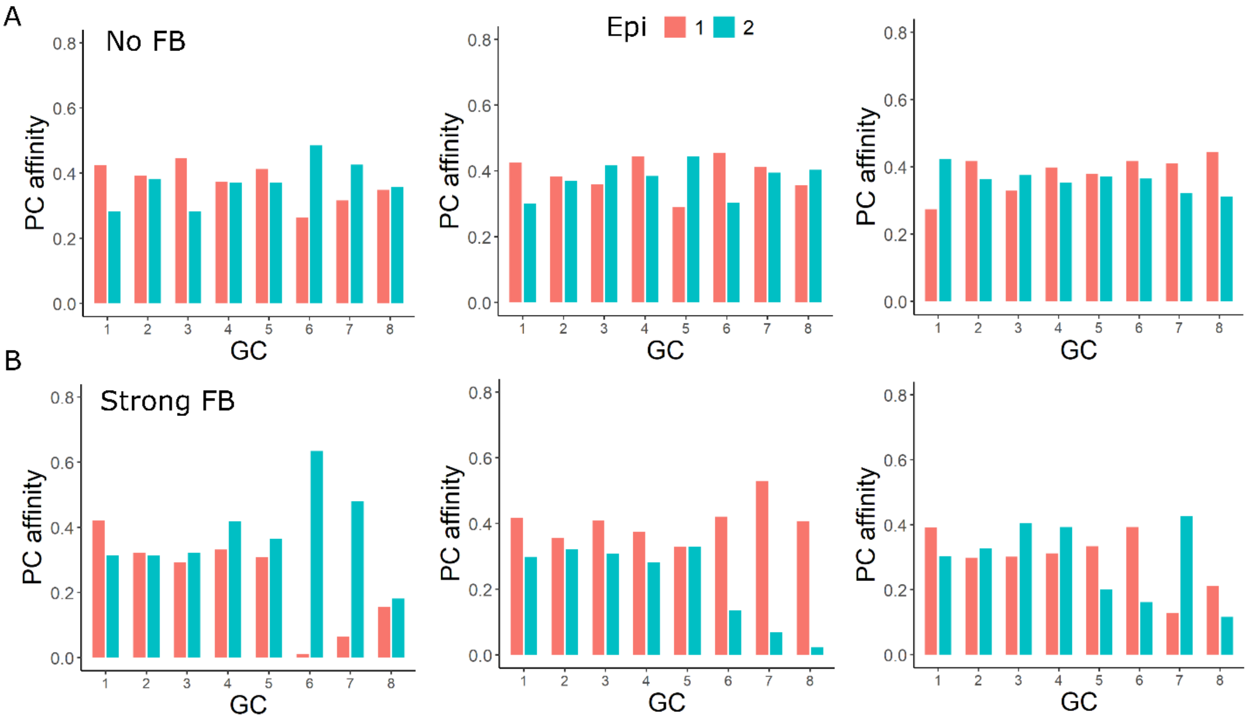

Figure S4: PC affinity towards two different epitopes. (A) with no interaction between GCs. B) with strong GC-GC interaction strength. Both epitopes (shape space positions 3333 and 5555) were considered in equal proportions and founder cells were chosen randomly anywhere in the shape space. GCs 1-8 were sorted in the sequence of initiation.

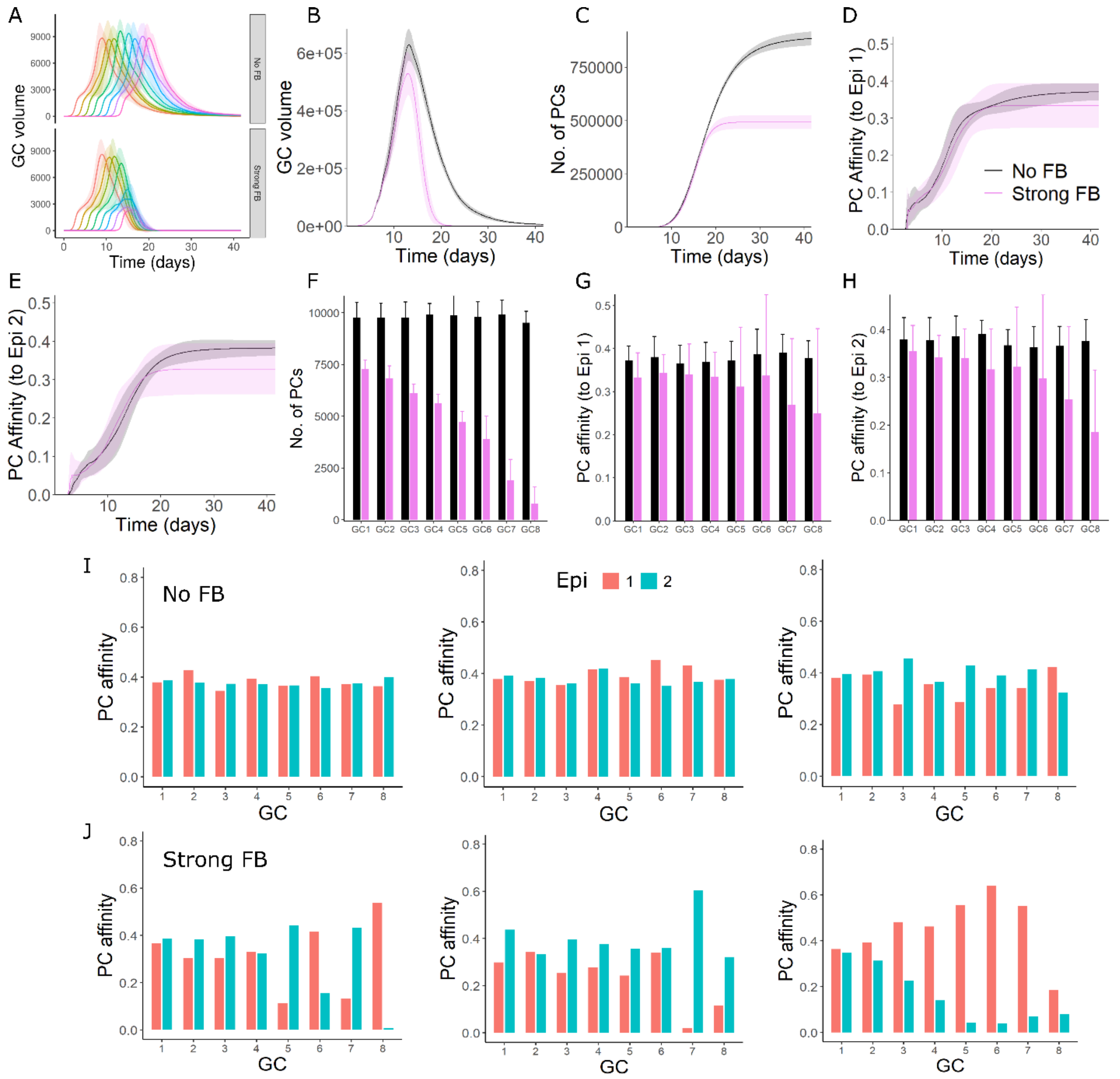

Figure S5: Impact of GC-GC interactions with two epitopes in equal proportions as in Figure S2 but with different shape space positions (3333 and 7777). A) Volume kinetics of simulated GCs. Color code represents the timing of GC initiation. B) Total volume of all GCs. C) Total plasma cell production of all GCs. D) Affinity of plasma cells from all GCs to Epi 1. E) Affinity of plasma cells from all GCs to Epi 2. Readouts in B-E represent the sum of corresponding readouts of all simulated GCs weighted by the number of non-explicitly simulated GCs in each case. F-H) Number of plasma cells, affinity of plasma cells to Epi 1 and 2 from each simulated GC. Inset in panel D shows the GC-GC interaction strength and applies for panels B-H. I and J) PC affinity towards two different epitopes with no interaction between GCs (I) and with strong GC-GC interaction strength (J). GCs were initialized asynchronously with two epitopes in equal proportion and founder cell positions chosen randomly anywhere in the shape space. GCs 1-8 were sorted in the sequence of initiation.

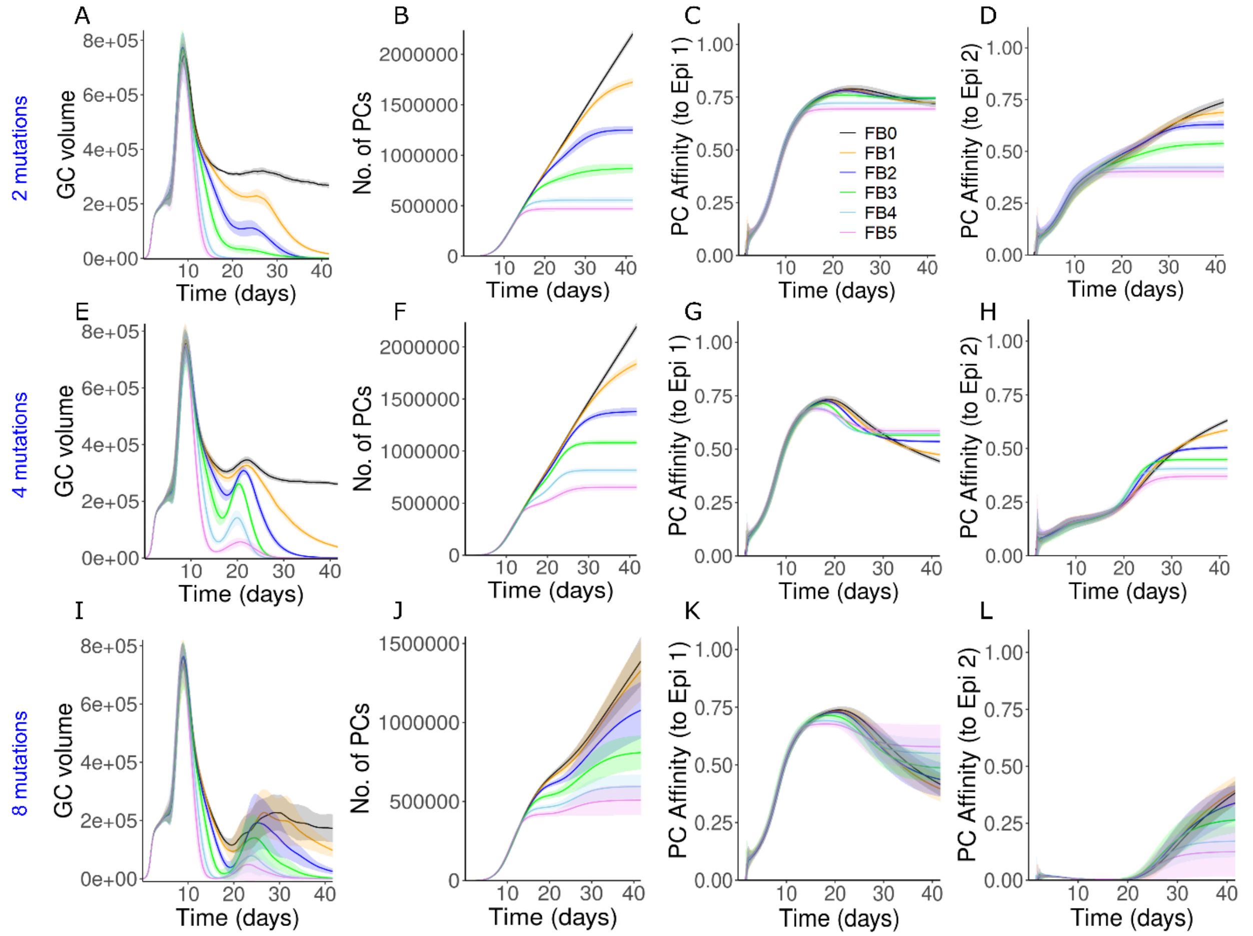

Figure S6: GC responses to antigenic variant (epitope 2) with different relatedness (2, 4 or 8 mutation distances in shape space) to epitope 1. A, E and I) Total volume of all GCs. B, F and J) Total plasma cell production of all GCs. C, G and K) Affinity of plasma cells from all GCs to epitope 1. D, H and L) Affinity of plasma cells from all GCs to epitope 2. Colors represent different GC-GC interaction strengths. FB0 represent simulations with no GC-GC interaction. FB1 and FB2 represent one-tenth and one-third of low interaction strength, respectively. FB3, FB4 and FB5 correspond to low, medium and strong interaction strengths, respectively. GCs were initialized with epitope 1 (shape space position 3333). Epitope 2 with mutation distance of 2 (panels A-D), 4 (panels E-H) or 8 (panels I-L) (shape space positions 3344, 4444 and 5555, respectively) from epitope 1 was added from day 7 of GC reaction. Founder cells of GCs were chosen randomly anywhere in the shape space. Inset in panel C applies for all panels.

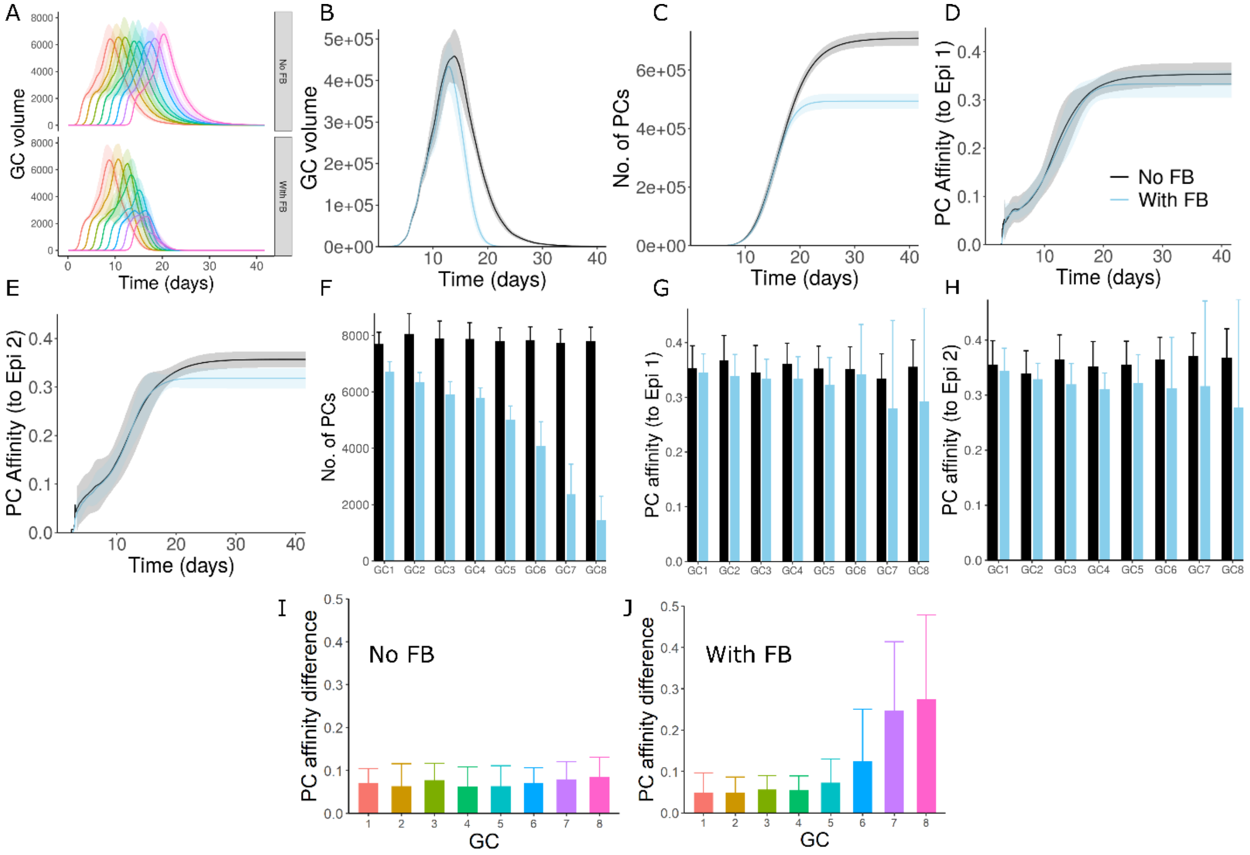

Figure S7: Impact of GC-GC interactions with two epitopes in equal proportions as in Figure S2 but with lower antigen concentration (2000 antigen portions). A) Volume kinetics of simulated GCs. Color code represents the timing of GC initiation. B) Total volume of all GCs. C) Total plasma cell production of all GCs. D) Affinity of plasma cells from all GCs to Epi 1. E) Affinity of plasma cells from all GCs to Epi 2. Readouts in B-E represent the sum of corresponding readouts of all simulated GCs weighted by the number of non-explicitly simulated GCs in each case. F-H) Number of plasma cells, affinity of plasma cells to Epi 1 and 2 from each simulated GC. Inset in panel D shows the GC-GC interaction strength and applies for panels B-H. I and J) Differences in affinity of PCs between two epitopes with no interaction between GCs (I) and with GC-GC interaction (J). GCs were initialized asynchronously with two epitopes in equal proportion and founder cell positions chosen randomly anywhere in the shape space. GCs 1-8 were sorted in the sequence of initiation. In simulations labelled "With FB", antibody production rate corresponding to medium GC-GC interaction strength (10^{-17} mol per cell per hour) was used.

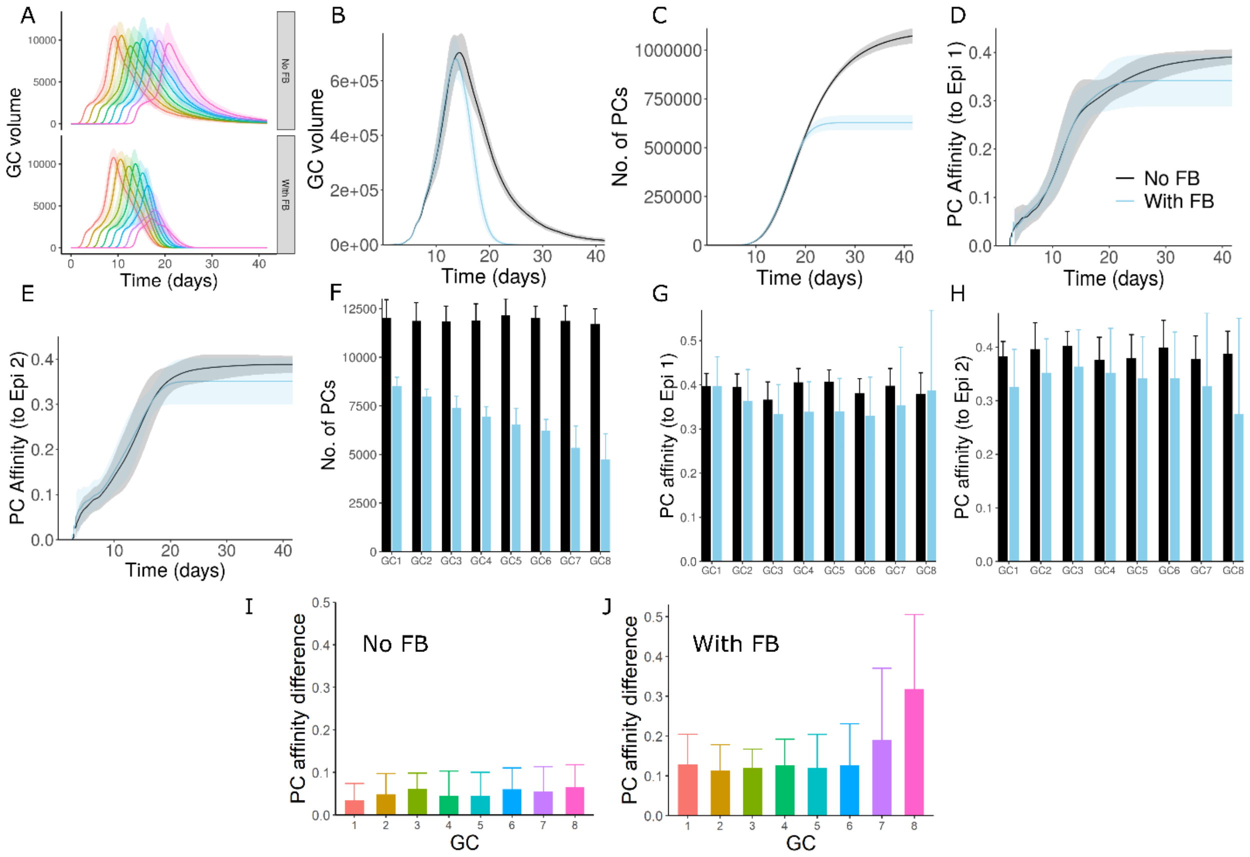

Figure S8: Impact of GC-GC interactions with two epitopes in equal proportions as in Figure S2 but with higher antigen concentration (4000 antigen portions). A) Volume kinetics of simulated GCs. Color code represents the timing of GC initiation. B) Total volume of all GCs. C) Total plasma cell production of all GCs. D) Affinity of plasma cells from all GCs to Epi 1. E) Affinity of plasma cells from all GCs to Epi 2. Readouts in B-E represent the sum of corresponding readouts of all simulated GCs weighted by the number of non-explicitly simulated GCs in each case. F-H) Number of plasma cells, affinity of plasma cells to Epi 1 and 2 from each simulated GC. Inset in panel D shows the GC-GC interaction strength and applies for panels B-H. I and J) Differences in affinity of PCs between two epitopes with no interaction between GCs (I) and with GC-GC interaction (J). GCs were initialized asynchronously with two epitopes in equal proportion and founder cell positions chosen randomly anywhere in the shape space. GCs 1-8 were sorted in the sequence of initiation. In simulations labelled "With FB", antibody production rate corresponding to medium GC-GC interaction strength (10^{-17} mol per cell per hour) was used.

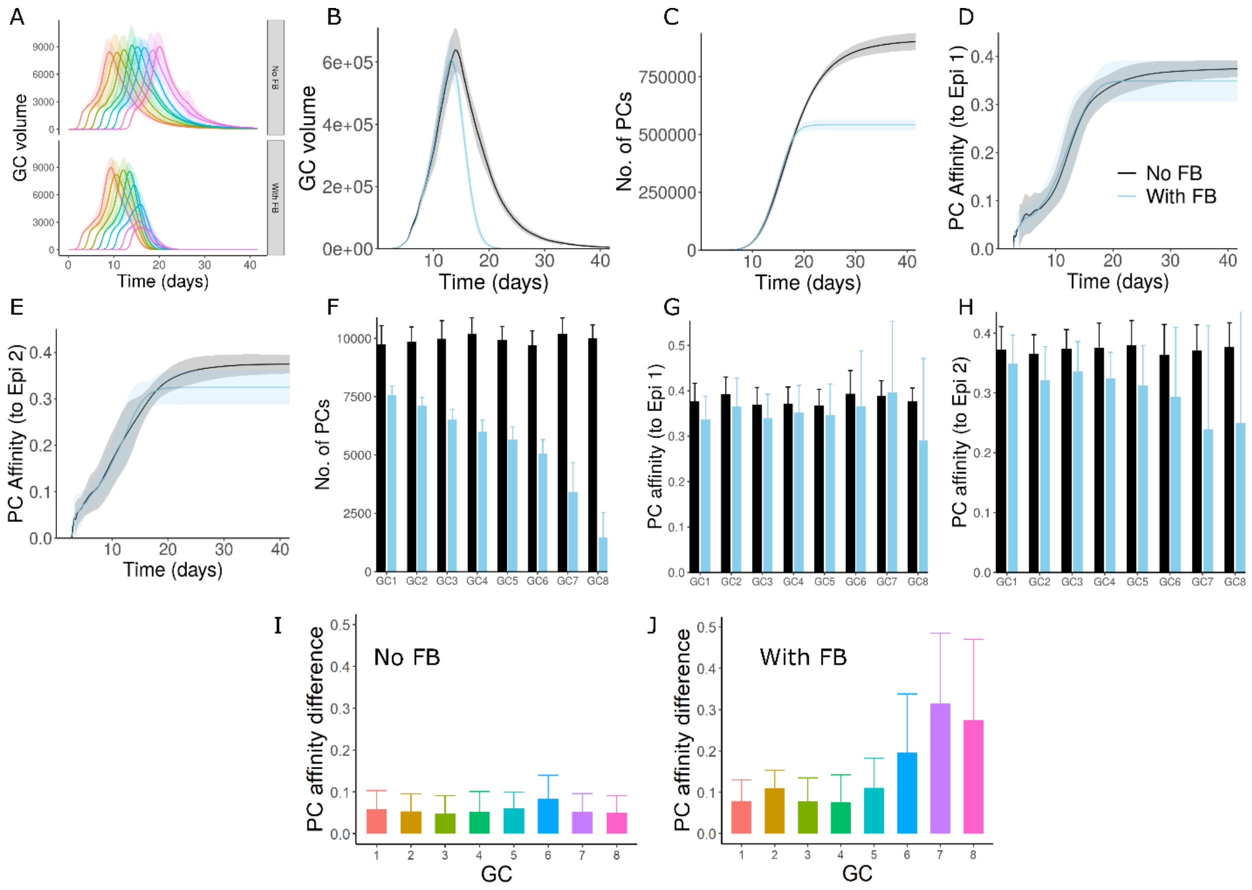

Figure S9: Impact of GC-GC interactions with two epitopes in equal proportions as in Figure S2 but with faster plasma cell differentiation corresponding to half-life of 12 days. A) Volume kinetics of simulated GCs. Color code represents the timing of GC initiation. B) Total volume of all GCs. C) Total plasma cell production of all GCs. D) Affinity of plasma cells from all GCs to Epi 1. E) Affinity of plasma cells from all GCs to Epi 2. Readouts in B-E represent the sum of corresponding readouts of all simulated GCs weighted by the number of non-explicitly simulated GCs in each case. F-H) Number of plasma cells, affinity of plasma cells to Epi 1 and 2 from each simulated GC. Inset in panel D shows the GC-GC interaction strength and applies for panels B-H. I and J) Differences in affinity of PCs between two epitopes with no interaction between GCs (I) and with GC-GC interaction (J). GCs were initialized asynchronously with two epitopes in equal proportion and founder cell positions chosen randomly anywhere in the shape space. GCs 1-8 were sorted in the sequence of initiation. In simulations labelled "With FB", antibody production rate corresponding to medium GC-GC interaction strength (10^{-17} mol per cell per hour) was used.

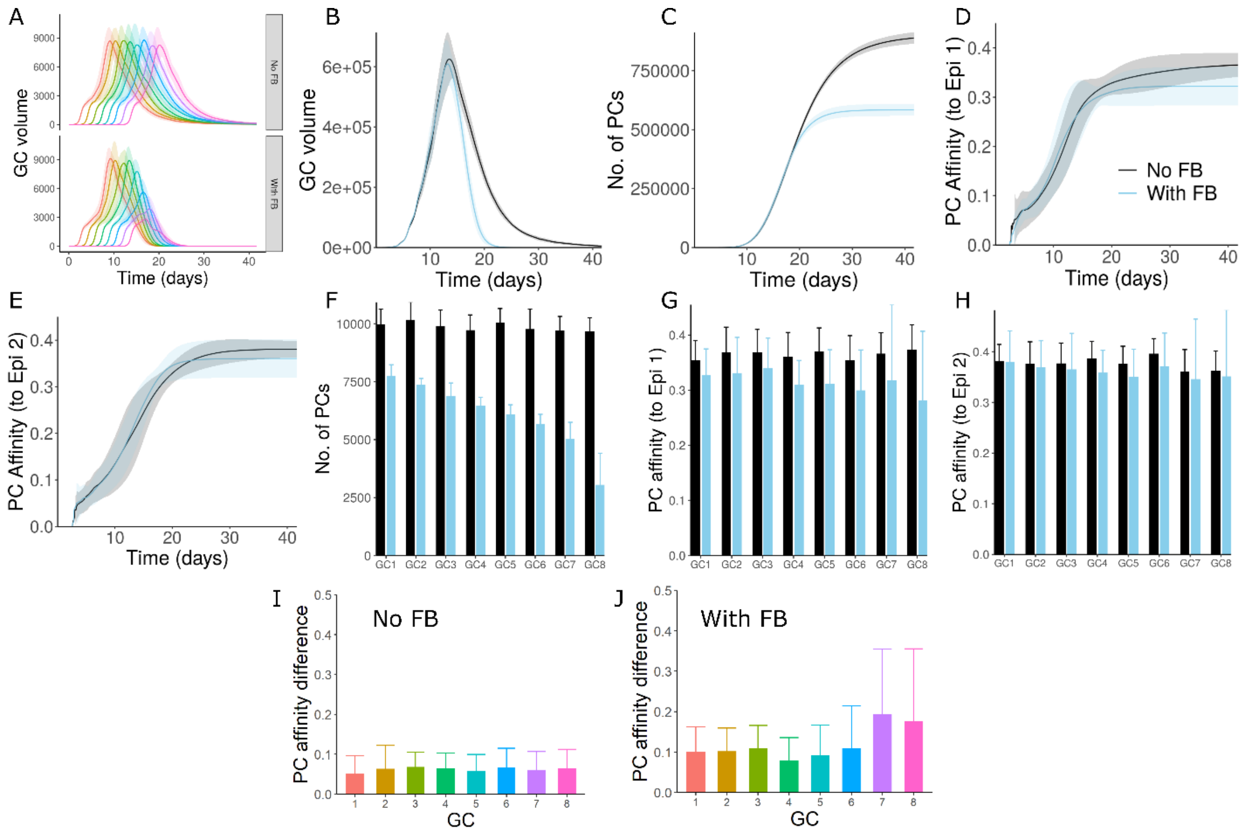

Figure S10: Impact of GC-GC interactions with two epitopes in equal proportions as in Figure S2 but with slower plasma cell differentiation corresponding to half-life of 48 days. A) Volume kinetics of simulated GCs. Color code represents the timing of GC initiation. B) Total volume of all GCs. C) Total plasma cell production of all GCs. D) Affinity of plasma cells from all GCs to Epi 1. E) Affinity of plasma cells from all GCs to Epi 2. Readouts in B-E represent the sum of corresponding readouts of all simulated GCs weighted by the number of non-explicitly simulated GCs in each case. F-H) Number of plasma cells, affinity of plasma cells to Epi 1 and 2 from each simulated GC. Inset in panel D shows the GC-GC interaction strength and applies for panels B-H. I and J) Differences in affinity of PCs between two epitopes with no interaction between GCs (I) and with GC-GC interaction (J). GCs were initialized asynchronously with two epitopes in equal proportion and founder cell positions chosen randomly anywhere in the shape space. GCs 1-8 were sorted in the sequence of initiation. In simulations labelled "With FB", antibody production rate corresponding to medium GC-GC interaction strength (10^{-17} mol per cell per hour) was used.

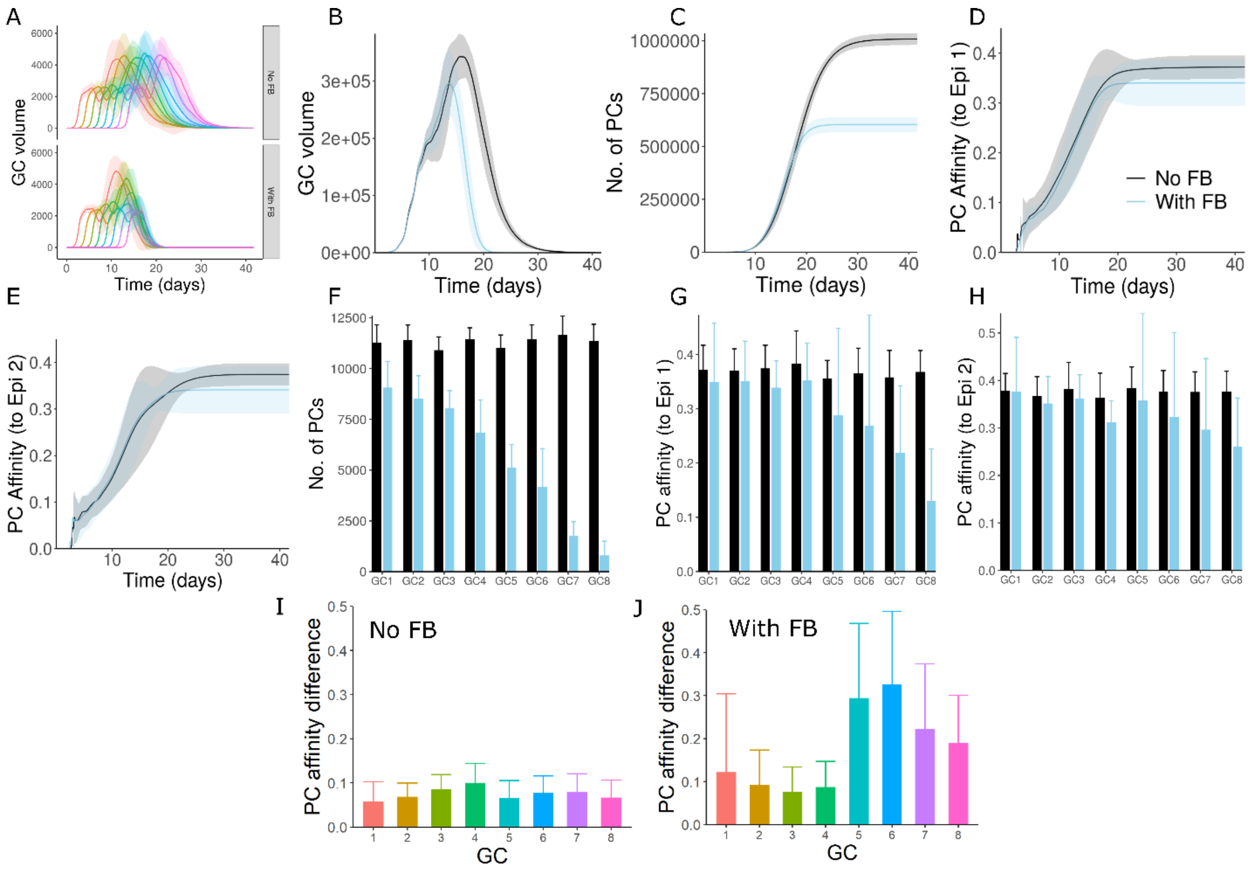

Figure S11: Impact of GC-GC interactions with two epitopes in equal proportions as in Figure S2 but with lower recycling probability (0.6). A) Volume kinetics of simulated GCs. Color code represents the timing of GC initiation. B) Total volume of all GCs. C) Total plasma cell production of all GCs. D) Affinity of plasma cells from all GCs to Epi 1. E) Affinity of plasma cells from all GCs to Epi 2. Readouts in B-E represent the sum of corresponding readouts of all simulated GCs weighted by the number of non-explicitly simulated GCs in each case. F-H) Number of plasma cells, affinity of plasma cells to Epi 1 and 2 from each simulated GC. Inset in panel D shows the GC-GC interaction strength and applies for panels B-H. I and J) Differences in affinity of PCs between two epitopes with no interaction between GCs (I) and with GC-GC interaction (J). GCs were initialized asynchronously with two epitopes in equal proportion and founder cell positions chosen randomly anywhere in the shape space. GCs 1-8 were sorted in the sequence of initiation. In simulations labelled “With FB”, antibody production rate corresponding to medium GC-GC interaction strength (10^{-17} mol per cell per hour) was used.

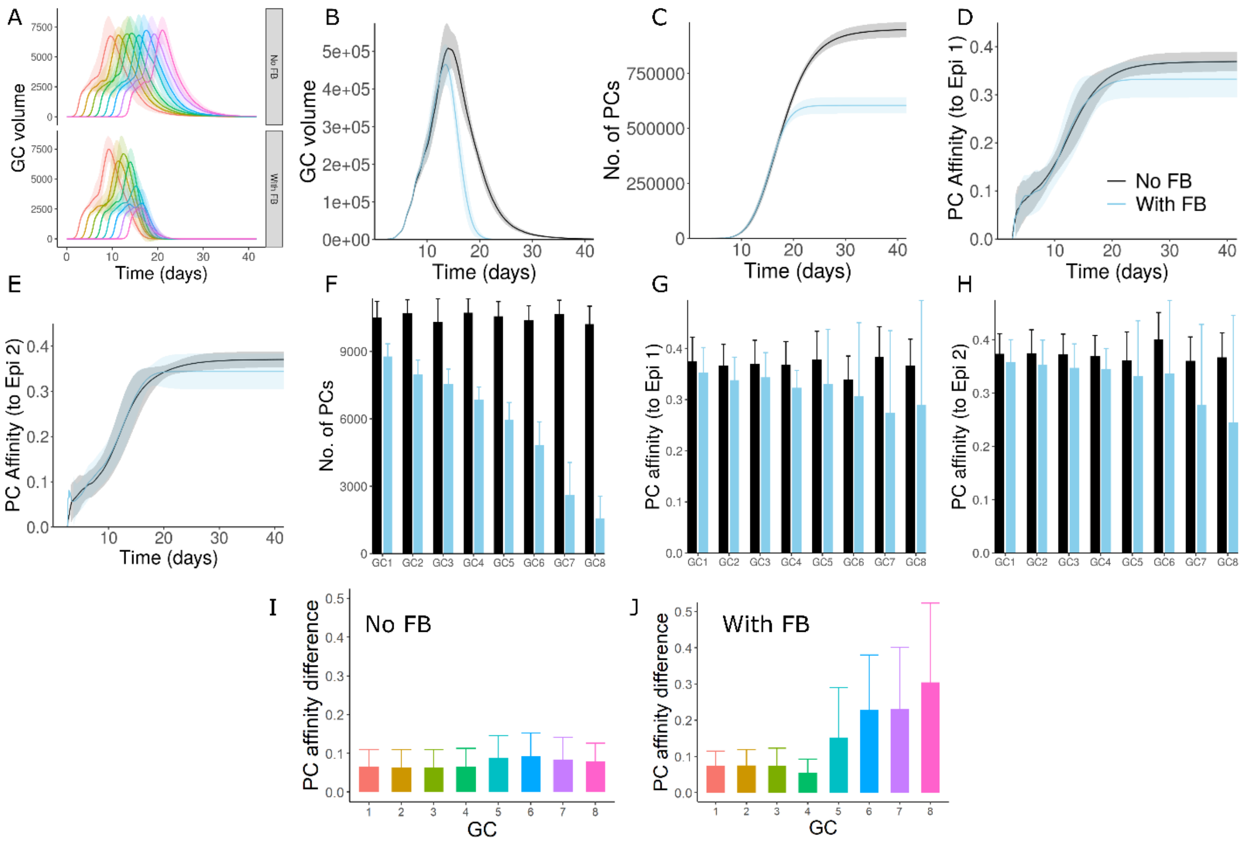

Figure S12: Impact of GC-GC interactions with two epitopes in equal proportions as in Figure S2 but with lower recycling probability (0.8). A) Volume kinetics of simulated GCs. Color code represents the timing of GC initiation. B) Total volume of all GCs. C) Total plasma cell production of all GCs. D) Affinity of plasma cells from all GCs to Epi 1. E) Affinity of plasma cells from all GCs to Epi 2. Readouts in B-E represent the sum of corresponding readouts of all simulated GCs weighted by the number of non-explicitly simulated GCs in each case. F-H) Number of plasma cells, affinity of plasma cells to Epi 1 and 2 from each simulated GC. Inset in panel D shows the GC-GC interaction strength and applies for panels B-H. I and J) Differences in affinity of PCs between two epitopes with no interaction between GCs (I) and with GC-GC interaction (J). GCs were initialized asynchronously with two epitopes in equal proportion and founder cell positions chosen randomly anywhere in the shape space. GCs 1-8 were sorted in the sequence of initiation. In simulations labelled "With FB", antibody production rate corresponding to medium GC-GC interaction strength (10^{-17} mol per cell per hour) was used.

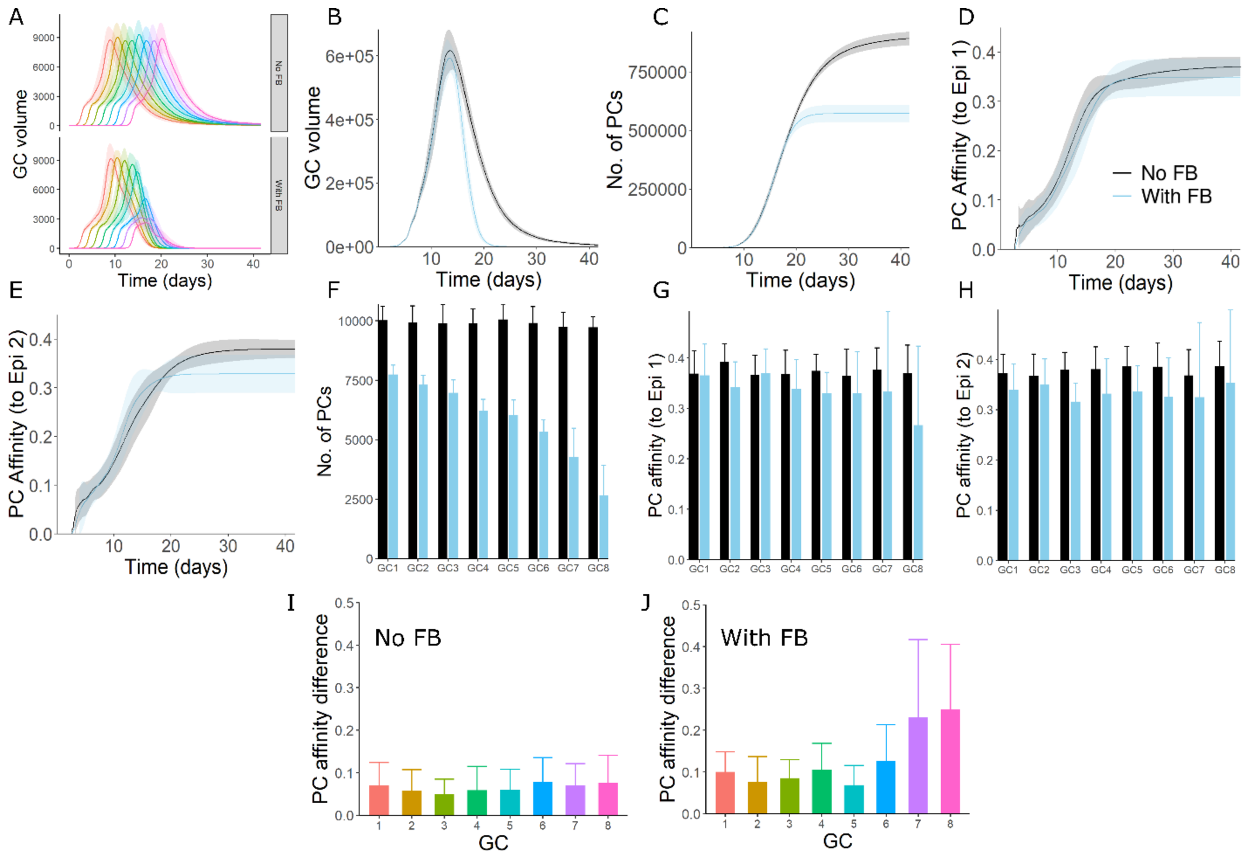

Figure S13: Impact of GC-GC interactions with two epitopes in equal proportions as in Figure S2 but with shorter antibody half-life (7 days). A) Volume kinetics of simulated GCs. Color code represents the timing of GC initiation. B) Total volume of all GCs. C) Total plasma cell production of all GCs. D) Affinity of plasma cells from all GCs to Epi 1. E) Affinity of plasma cells from all GCs to Epi 2. Readouts in B-E represent the sum of corresponding readouts of all simulated GCs weighted by the number of non-explicitly simulated GCs in each case. F-H) Number of plasma cells, affinity of plasma cells to Epi 1 and 2 from each simulated GC. Inset in panel D shows the GC-GC interaction strength and applies for panels B-H. I and J) Differences in affinity of PCs between two epitopes with no interaction between GCs (I) and with GC-GC interaction (J). GCs were initialized asynchronously with two epitopes in equal proportion and founder cell positions chosen randomly anywhere in the shape space. GCs 1-8 were sorted in the sequence of initiation. In simulations labelled “With FB”, antibody production rate corresponding to medium GC-GC interaction strength (10^{-17} mol per cell per hour) was used.

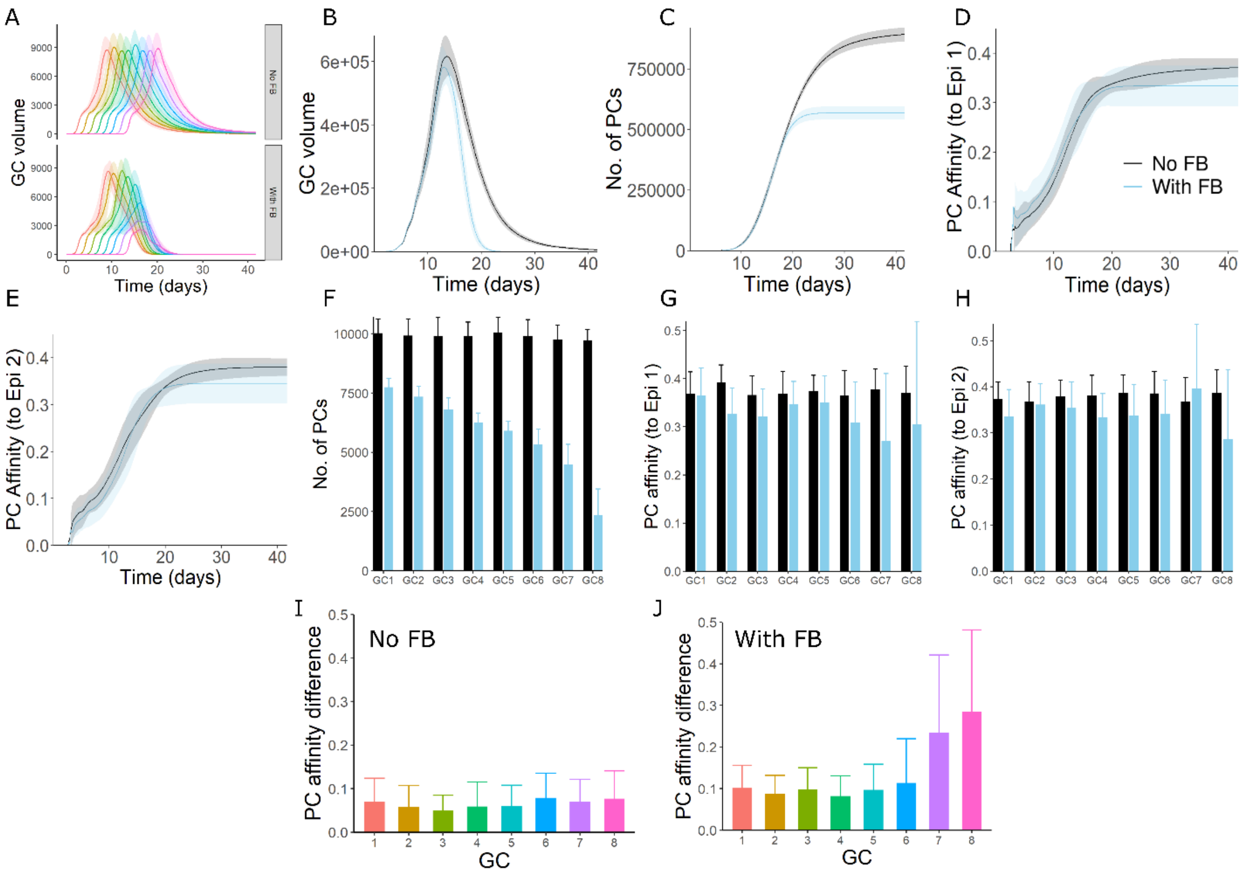

Figure S14: Impact of GC-GC interactions with two epitopes in equal proportions as in Figure S2 but with longer antibody half-life (21 days). A) Volume kinetics of simulated GCs. Color code represents the timing of GC initiation. B) Total volume of all GCs. C) Total plasma cell production of all GCs. D) Affinity of plasma cells from all GCs to Epi 1. E) Affinity of plasma cells from all GCs to Epi 2. Readouts in B-E represent the sum of corresponding readouts of all simulated GCs weighted by the number of non-explicitly simulated GCs in each case. F-H) Number of plasma cells, affinity of plasma cells to Epi 1 and 2 from each simulated GC. Inset in panel D shows the GC-GC interaction strength and applies for panels B-H. I and J) Differences in affinity of PCs between two epitopes with no interaction between GCs (I) and with GC-GC interaction (J). GCs were initialized asynchronously with two epitopes in equal proportion and founder cell positions chosen randomly anywhere in the shape space. GCs 1-8 were sorted in the sequence of initiation. In simulations labelled “With FB”, antibody production rate corresponding to medium GC-GC interaction strength (10^{-17} mol per cell per hour) was used.

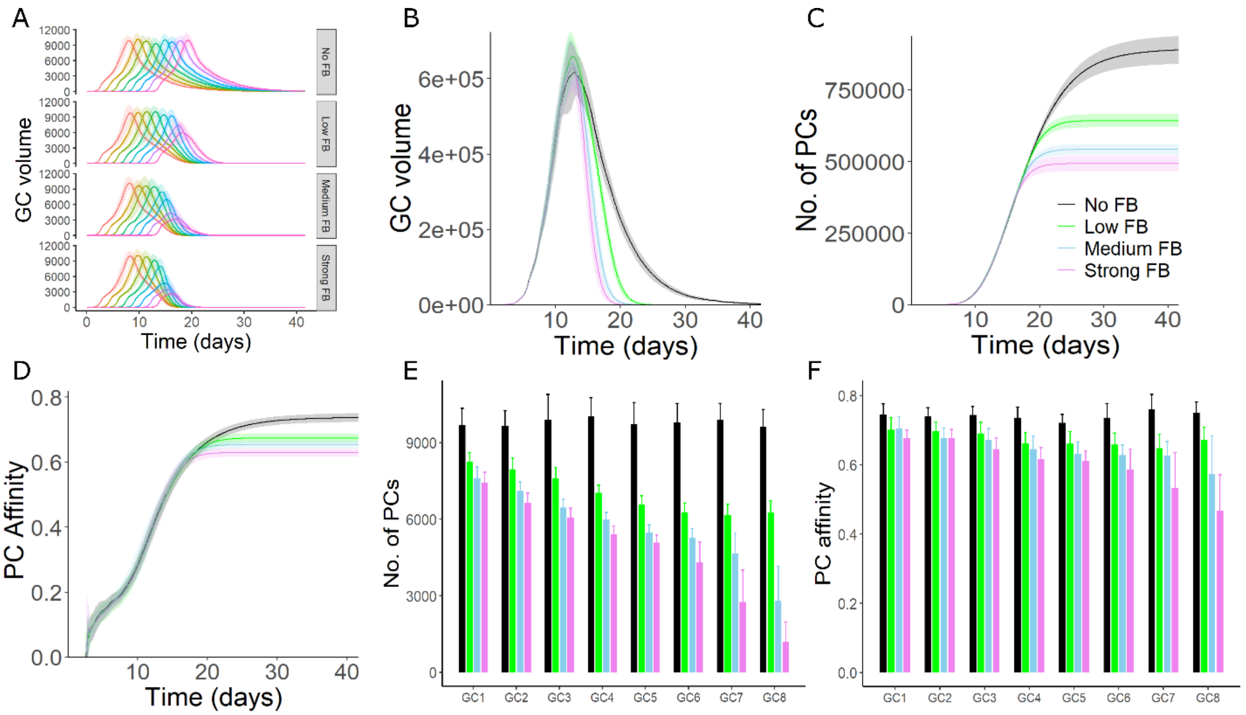

Figure S15: Effect of GC-GC interactions as in Figure 1 but with different seeder affinities. A) Volume kinetics of simulated GCs. GCs were initialized asynchronously and color code represents the timing of GC initiation. B) Total volume of all GCs. C) Plasma cell production from all GCs. D) Affinity of plasma cells from all GCs. Readouts in B-D represent the sum of corresponding readouts of all simulated GCs weighted by the number of non-explicitly simulated GCs in each case. E) Number of plasma cells produced from individual GCs. F) Affinity of plasma cells from individual GCs. GCs 1-8 were sorted in the sequence of initiation. Inset in panel C shows the GC-GC interaction strength (see methods) and applies for panels B-F. A single epitope (shape space position 3333) was considered with founder cell positions randomly chosen between 1-8 mutations from the epitope in the shape space.
